# Eating habits during quarantine: Investigating the role of emotions and loneliness in a sample of adults in Greece

**DOI:** 10.1192/j.eurpsy.2022.1364

**Published:** 2022-09-01

**Authors:** N. Savvopoulou, K. Assimakopoulos, P. Gourzis, E. Jelastopulu

**Affiliations:** 1 UNIVERSITY OF PATRAS, Department Of Public Health, Medical School, PATRAS, Greece; 2 UNIVERSITY OF PATRAS, Department Of Psychiatry, Medical School, PATRAS, Greece; 3 General University Hospital of Patras, Greece, Department Of Psychiatry, Patras, Greece; 4 School of Medicine, University of Patras, Public Health, Rio, Greece

**Keywords:** Loneliness, quarantine, eating habits, Emotions

## Abstract

**Introduction:**

Imposing quarantine as a measure to manage the coronavirus pandemic is a stressful event that is often associated with negative psychological effects. Eating habits seemed to be significantly affected during the quarantine, while strong negative emotions were triggered as the feeling of loneliness increased at the same time.

**Objectives:**

This study aims to investigate the eating habits of individuals during quarantine and the role of positive and negative emotions and loneliness in shaping these habits.

**Methods:**

An online cross-sectional study was performed using 3 validated scales, EAT-26 (3 subscales: Dieting, Bulimia and Food Preoccupation, Oral Control), Modified Differential Emotions Scale and UCLA Loneliness Scale. Data was collected between April and May 2021 mainly from social media platforms. Statistical analyses included linear regression and mediation analyses.

**Results:**

Abnormal eating habits were detected in 25% of the participants (N= 450, ages 18-74) while the majority reported medium rates of negative/positive emotions and loneliness. Female sex is associated with abnormal eating habits (p=0.010) and mainly dietary behaviors (p=0.029). Negative emotions (p=0.032) and loneliness (p=0.001) seem to be predictive factors of eating habits in general and bulimic behaviors. Negative emotions correlate directly with eating habits. However, we found a significant mediation of loneliness (p=0.032). Furthermore, the observed association between negative emotions and bulimia is partly mediated by loneliness (p=0.018).

**Conclusions:**

Negative emotions and loneliness seemed to play an important role in shaping eating habits during quarantine. Multilevel public health interventions are needed to address the negative effects of quarantine and pandemic in general.

**Disclosure:**

No significant relationships.

